# Attenuation of Pancreatic Cancer In Vitro and In Vivo via Modulation of Nrf2 and NF-κB Signaling Pathways by Natural Compounds

**DOI:** 10.3390/cells10123556

**Published:** 2021-12-16

**Authors:** Marta Cykowiak, Robert Kleszcz, Małgorzata Kucińska, Jarosław Paluszczak, Hanna Szaefer, Adam Plewiński, Hanna Piotrowska-Kempisty, Marek Murias, Violetta Krajka-Kuźniak

**Affiliations:** 1 Department of Pharmaceutical Biochemistry, Poznan University of Medical Sciences, 4, Święcickiego Street, 60-781 Poznań, Poland; marta.cykowiak@ump.edu.pl (M.C.); kleszcz@ump.edu.pl (R.K.); paluszcz@ump.edu.pl (J.P.); hszaefer@ump.edu.pl (H.S.); 2 Department of Toxicology, Poznan University of Medical Sciences, 30, Dojazd Street, 60-631 Poznań, Poland; kucinska@ump.edu.pl (M.K.); hpiotrow@ump.edu.pl (H.P.-K.); marek.murias@ump.edu.pl (M.M.); 3 Centre for Advanced Technologies, Adam Mickiewicz University, 10, Uniwersytetu Poznańskiego Street, 61-614 Poznań, Poland; adam.plewinski@amu.edu.pl

**Keywords:** canonical and non-canonical Nrf2 pathway, NF-κB pathway, phytochemicals, PSN-1 cells, MS1 cells, mice xenograft model

## Abstract

Pancreatic cancer is a disease in which deregulation of signaling pathways plays a key role, thus searching for their novel modulators is a promising therapeutic strategy. Hence, in this study, the effect of phytochemical combinations on the canonical and non-canonical activation of Nrf2 and its interaction with the NF-κB pathway was evaluated in extensively proliferating pancreatic cancer cell line, PSN-1, in comparison to non-cancerous MS1 cells. The activation of Nrf2 and NF-κB, expression of their target genes, and effect on cell survival were assessed in PSN-1 cells. The tumor burden was evaluated in mice carrying xenografts. PSN-1 cells were more sensitive to the tested compounds as compared to the MS1 cell line. Combination of xanthohumol and phenethyl isothiocyanate was more effective than single compounds at decreasing the canonical and non-canonical activation of Nrf2 in PSN-1 cancer cells. Decreased activation of NF-κB, and subsequent reduced cytosolic COX-2 and nuclear STAT3 level indicated their anti-inflammatory and pro-apoptotic activities. In vivo studies showed the partial response in groups treated with xanthohumol or the combination of xanthohumol and phenethyl isothiocyanate. Overall, these results suggest that the combination of xanthohumol and phenethyl isothiocyanate may be a promising therapeutic candidate against pancreatic cancer.

## 1. Introduction

Maintenance of homeostasis at the cellular level, in terms of various external and internal stimuli permanently influencing the human body, requires the key transcription factors. One of them is the nuclear factor-erythroid 2related factor 2 (Nrf2). Nrf2 orchestrates the expression of genes involved in the regulation of reduction-oxidation processes, metabolism of xenobiotics, autophagy, inflammation, DNA repair, and processes related to immune response [1,2,3]. The activation of Nrf2 is caused by reactive oxygen species (ROS), synthetic compounds, as well as several phytochemicals [4,5].

To date, the canonical and non-canonical mechanisms of the Nrf2 activation have been described [6]. Under normal conditions, Nrf2 is present in the cytoplasm associated with its inhibitor protein—Kelch-like ECH-associated protein 1 (Keap1). When cells encounter oxidative stress or Nrf2 activators, Nrf2 quickly disconnects from Keap1 leading to the inhibition of Nrf2 degradation [7]. Subsequently, Nrf2 translocates to the nucleus and regulates anti-oxidative stress response by binding to the antioxidant response elements (AREs) and activating the expression of cytoprotective genes [8].

In the non-canonical pathway, the essential role is played by p62/sequestosome 1 (SQSTM1), an autophagy adaptor, which promotes Nrf2 nuclear translocation and activates ARE-related genes [9]. Moreover, it has been demonstrated that the interaction between p62 and Keap1 enables p62 to sequester Keap1 into autophagosomes and abrogate the ubiquitin-proteasome degradation of Nrf2, preventing Nrf2 activation [8,10].

Prolonged oxidative stress can lead to the activation of the inflammatory response, mainly triggered by transcription factors such as NF-κB, which plays a major role in the development, maintenance, and progression of most chronic diseases.

The activation of NF-κB by various inducers such as cytokines and ROS leads to the translocation of NF-κB from the cytoplasm to the nucleus and subsequent initiation of the expression of a wide variety of genes encoding pro-inflammatory proteins [11,12]. As an analog of NF-κB, Nrf2 plays an essential role in cellular antioxidant defense mechanisms [13] and a protective role against inflammation and responds to pro-inflammatory stimuli. Thus, the cells are protected by Nrf2 from inflammatory injuries via modulating cellular defense against oxidative stress and electrophilic insults by phase-II detoxifying and antioxidative enzymes [14].

Several studies have shown the activation of Nrf2 by phytochemicals in in vitro and in vivo models [15]. According to recent reports, the combination of phytochemicals can act more effectively than monotherapy [16,17]. Xanthohumol (XN, a prenylated chalcone from hop plant) [18], phenethyl isothiocyanate (PEITC, the product of glucosinolates degradation, found in cruciferous plants) [19], indole-3-carbinol (I3C, the product of glucosinolates degradation, found in cruciferous plants) [19], and resveratrol (RVS, a stilbene derivative, the component of grape skin) [20] have been revealed to modulate the Nrf2 signaling pathway in vitro. Our recent studies have demonstrated that xanthohumol, phenethyl isothiocyanate, resveratrol, indole-3-carbinol, and their combinations are able to influence the activation of Nrf2 by translocation of Nrf2 from the cytosol to the nuclear compartment and subsequently increased expression of Nrf2 and antioxidant and detoxifying genes in PANC-1 [21] and Mia-Pa-Ca-2 pancreatic cancer cell lines [17].

Pancreatic cancer is the fourth leading cause of cancer-related deaths worldwide. The overall five-year survival rate for this malignancy remains the lowest of any cancer at around 7%, due to limited diagnostic methods, disease aggressiveness, and a lack of targeted therapeutic interventions [22]. As its incidence has been increasing in recent years, the search for new and more effective treatments becomes even more urgent. The most common type of pancreatic cancer is adenocarcinoma. Thus, in the current study, we used PSN-1 pancreatic adenocarcinoma cells. In our previous studies, we used epithelioid carcinoma cell lines—PANC-1 and Mia-Pa-Ca-2 [17,21].

In order to continue and to study in detail how xanthohumol, phenethyl isothiocyanate, resveratrol, indole-3-carbinol, and their combinations modulate the Nrf2 pathway, and how this modulation can correspond with the NF-κB signaling pathway, we focused on the evaluation of the effect of these phytochemicals on Nrf2 canonical and non-canonical pathway activation, as well as the interaction between Nrf2 and NF-κB, and STATs signaling pathways in extensively proliferating pancreatic cancer cell line—PSN-1 and tumor growth in vivo. 

## 2. Materials and Methods

### 2.1. Materials and Chemicals

All of the analyzed compounds: PEITC, I3C, XN, and RVS with purity over 98% were purchased from Sigma Aldrich (St. Louis, MO, USA). Roswell Park Memorial Institute-1640 (RPMI-1640) Medium, Dulbecco’s modified Eagle’s medium (DMEM), antibiotics solution (10^4^ U penicillin, 25 μg amphotericin B, 10 mg streptomycin in 100 mL), dimethyl sulfoxide (DMSO), fetal bovine serum (FBS), and Tris were obtained from Sigma Aldrich (St. Louis, MO, USA). Primary antibodies against Nrf2, SOD, NQO1, Keap1, GSK-3β, phosphorylated-GSK-3β, NF-κB p50, NF-κB p65, COX-2, β-actin, and lamin were obtained from Santa Cruz Biotechnology (Santa Cruz, CA, USA); antibody against p62 from Cell Signaling Technology (Danvers, MA, USA), an antibody against GSTP from LabAs (Tartu, Estonia). All HRP and AP-labeled secondary antibodies were provided by Santa Cruz Biotechnology (Santa Cruz, CA, USA). All the other chemicals were commercial products of the highest purity available.

### 2.2. Cell Culture, Cell Viability Assay, and Treatment with Tested Compounds

**Figure 1:**
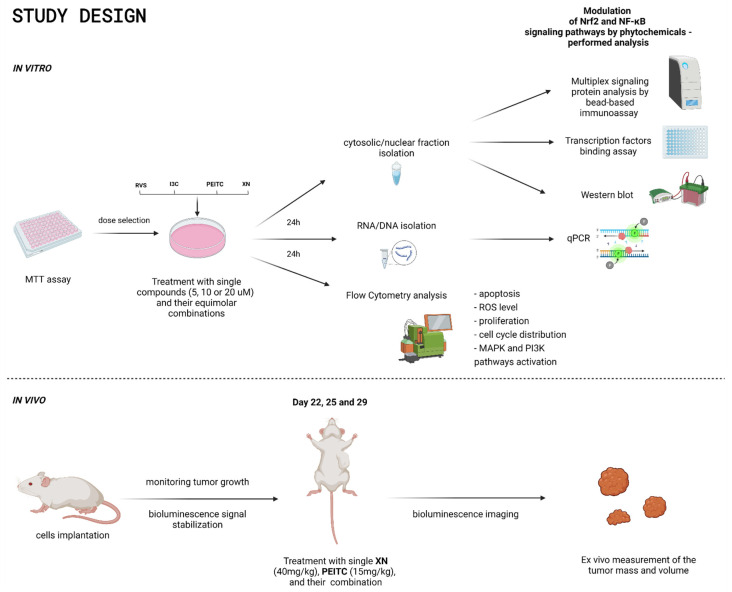
The scheme of study design.

The study design is presented in Figure 1. The human pancreatic cancer cell line PSN-1 (ATCC, Manassas, VA, USA) was maintained in RPMI-1640 and a mouse-derived pancreatic islet endothelial cell line, MS1 (ATCC, Manassas, VA, USA) cell line in DMEM. Both media were supplemented with 10% FBS and 1% antibiotics solution. The cell lines were cultured in standard conditions (37 °C, 5% CO_2_). To assess the cytotoxic activity of the analyzed phytochemicals: I3C, PEITC, XN, RVS, and their combinations, the MTT assay was used according to standard protocols. PSN-1 and MS1 cells were seeded in 96-well plates at the density of 10^4^ cells per well and maintained in a complete growth medium. The tested natural compounds and their combinations were added to the culture medium at the concentrations range from 0.5–150 µM and incubated for 24 h. Next, the cells were washed with PBS and incubated with a growth medium containing MTT salt (0.5 mg/mL) for 4 h. The precipitated formazan crystals were dissolved in acidic isopropanol and the absorbance was measured at 540 and 690 nm [23]. The experiment was performed in triplicate. Based on the MTT assay we selected the concentrations of 5, 10, and 20 µM for in vitro assays. These concentrations did not exceed values assuring 70% of viability for cancer and non-cancerous cells. The combinations of phytochemicals were prepared in equimolar concentrations: for 5 µM, 2.5 µM of each compound; for 10 µM, 5 µM of each compound; and for 20 µM, 10µM of each compound. The higher doses of compounds and combinations—10 µM and 20 µM—were used in preliminary flow cytometry analysis. Since in the measurement of apoptosis we confirmed an increased level of apoptotic cells caused by XN 20 µM in non-cancerous MS1 cell line, we decided to exclude this concentration in further analysis. The doses for in vivo studies have been selected after careful analysis of available literature data [24,25,26,27]. To measure the effect of the evaluated compounds on the chosen parameters, 10^6^ cells were seeded per 100 mm diameter culture dish and subjected to an initial 24-h incubation. After incubation, the cells were treated with all of the tested phytochemicals alone at the concentrations of 5, 10, and 20 µM or their equimolar mixture for 24 h and harvested. We used DMSO as vehicle control. 

### 2.3. Quantitative Real-Time PCR 

Total RNA was extracted from PSN-1 and MS1 cells using the GeneMatrix Universal DNA/RNA/Protein Purification Kit (EurX, Gdańsk, Poland) according to the manufacturer’s protocol. For total RNA reverse transcription, the Revert-Aid First Strand cDNA Synthesis kit (Fermentas, Burlington, ON, Canada) was used. Quantitative real-time analyses were conducted using the Maxima SYBR Green Kit (Fermentas, Burlington, ON, Canada) and BioRad Chromo4 (Hercules, CA, USA) or Roche LightCycler96 (Pleasanton, CA, USA) thermal cyclers. The protocol started with 5 min of enzyme activation at 95 °C, followed by 40 cycles of 95 °C for 15 s, 56 °C for 20 s, and 72 °C for 40 s, and final elongation at 72 °C for 5 min [18]. The melting curve was used to verify the specificity of the products. The expression of *TBP* (TATA box-binding protein) and *PBGD* (porphobilinogen deaminase) reference genes were used for normalization. All primers were designed by Beacon Designer software and BLAST searched to minimize unspecific binding. We selected primer pairs generating intron-spanning amplicons. The primers were obtained from the Institute of Biochemistry and Biophysics, Polish Academy of Sciences (Warsaw, Poland). Primer sequences are presented in Table 1.

### 2.4. Transcription Factors Binding Assay

To assess Nrf2 and NF-κB activation, enzymatic immunoassays were performed using the Transcription Factor ELISA Assay Kits (TransAM™Nrf2/TransAM™NF-κB, Active Motif, Carlsbad, CA, USA) as previously described [21]. Nuclear fractions, isolated using the Nuclear/Cytosol Fractionation Kit (BioVision Research, Milpitas, CA, USA) were added to the oligonucleotide-coated wells, where the oligonucleotide sequence contained the Nrf2/ARE (5′-GTCACAGTGACTCAGCAGAATCTG-3′) or the NF-κB (5′-GGGACTTTCC-3′) consensus binding site, respectively. The wells were then washed, and the antibody against Nrf2 or NF-κB was added. Subsequent addition of an HRP-conjugated secondary antibody provided a sensitive colorimetric readout at 450 nm.

### 2.5. Western Blot

We determined the protein level of the analyzed parameters using the Western blot assay according to the previously published protocol [18]. Briefly, the cytosolic and nuclear extracts were prepared from the cells using the Nuclear/Cytosol Fractionation Kit (BioVision Research, Milpitas, CA, USA). Nuclear (Nrf2, NF-κB p50, NF-κB p65, lamin) or cytosolic proteins (Nrf2, Keap1, p62, p-GSK3β, GSK3β, GSTP, SOD, NQO1, NF-κB subunits p50 and p65, COX-2, β-actin) (50–100 µg) were separated on 12% or 10% SDS-PAGE slab gels, and proteins were transferred onto nitrocellulose membranes. After blocking with 10% skimmed milk, the proteins were probed with specific antibodies. As an internal control, we used β-actin or lamin. Alkaline phosphatase-labeled or horseradish peroxidase-labeled anti-goat IgG, anti-rabbit IgG, and anti-mouse IgG were used as secondary antibodies in the staining reaction. The amount of immunoreactive products in each lane was determined using the Chemidoc^TM^ Touch Imaging System with Image Lab software (BioRad Laboratories, Hercules, CA, USA).

### 2.6. Multiplex Signaling Protein Analysis by Bead-Based Immunoassay

Magnetic bead-based immunoassay was performed on the Luminex MAGPIX^®^ multiplex immunoassay system. Data were analyzed with Milliplex Analyst 5.1 (EMD Millipore, Burlington, MA, USA). The panel we performed included quantitation of the following proteins in the cytosolic fractions of PSN-1 cells— STAT3, STAT5, CREB, p70S6K, ERK1/2, AKT, p38, and JNK—according to the manufacturer’s instructions. The magnetic bead panel with high sensitivity antibodies against analyzed proteins was obtained from Merck (Darmstadt, Germany). A multiplex test based on microspheres using Luminex^®^ xMAP^®^ technology with different fluorescent colors was conducted using a compatible MAGPIX^®^ camera. Cytosolic fractions were suspended in MILLIPLEX^®^ MAP buffer. The beads suspension was placed in each well of a 96-well plate, samples were added into the wells and incubated overnight at 2–8 °C on a shaker, protected from light. The plate was washed with 2× buffer, and then 1× MILLIPLEX^®^ MAP Detection Antibodies were added. After shaking for 1 h at room temperature, the antibodies were removed, and 1× MILLIPLEX^®^ MAP Streptavidin/Phycoerythrin (SAPE) was added. Then the MILLIPLEX^®^ MAP Amplification Buffer was added to each well and plates were shaken for 15 min. The beads were suspended in MILLIPLEX^®^ MAP buffer, and each microsphere was identified with a MAGPIX^®^ Luminex Analyzer (Merck, Darmstadt, Germany), and the results were calculated from the reporters’ fluorescent signals. Mean fluorescence intensities were quantified with the xPonent 4.2 software (Luminex Corporation, Austin, TX, USA). The raw MFI results for the tested protein levels were converted relative to control (DMSO-treated cells).

### 2.7. Flow Cytometry Cell Analysis

#### 2.7.1. Cell Cycle Distribution

The Muse^®^ Cell Cycle Kit (Merck, Darmstadt, Germany) was used in the determination of cell cycle according to the manufacturer’s protocol. The cell cycle distribution analysis was performed by propidium iodide staining and its specificity in binding to DNA was improved by the addition of RNase A. Briefly, cells (3 × 10^5^ per well) were seeded in 6-well plates, pre-incubated for 24 h, and further grown for 24 h in the presence of the tested compounds. Topotecan (0.1 µM) treated cells were used as a positive control of the cell cycle arrest. Subsequently, cells were harvested, fixed in ice-cold 70% ethanol, and frozen until further analysis at –20 °C. The next day, fixed cells were collected, washed with PBS buffer, and stained with the mixture of reagents 30 min before flow cytometry analysis on Muse^®^ Cell Analyzer. Data (Merck, Darmstadt, Germany) were evaluated using Muse^®^ Analysis Software ver. 1.5 (Merck, Darmstadt, Germany).

#### 2.7.2. Cell Proliferation

The expression of Ki67 protein was analyzed using the Muse^®^ Ki67 Proliferation Kit (Merck, Darmstadt, Germany) according to the manufacturer’s protocol. Briefly, cells (3 × 10^5^ per well) were seeded in 6-well plates, pre-incubated for 24 h, and further grown for 24 h in the presence of the tested compounds. As a positive control of the anti-proliferative effect, we used starved cells (cultured in medium without FBS). Next, cells were harvested, fixed, and exposed to permeabilization buffer with subsequent incubation with the Muse^®^ Hu Ki67 Antibody for 30 min in the dark at room temperature. Cells were analyzed by flow cytometry on Muse^®^ Cell Analyzer, and data were evaluated using Muse^®^ Analysis Software ver. 1.5 (Merck, Darmstadt, Germany).

#### 2.7.3. Apoptosis

The Muse^®^ Annexin V and Dead Cell Kit (Merck, Darmstadt, Germany) was used in the evaluation of the apoptosis according to the manufacturer’s protocol, using one of the known apoptosis markers—the externalization of phosphatidylserine. Additionally, the 7-Aminoactinomycin D (7-AAD) staining enables discrimination between early and late apoptotic cells. Briefly, cells (3 × 10^5^ per well) were seeded in 6-well plates, pre-incubated for 24 h, and further grown for 24 h in the presence of the tested compounds and Topotecan (0.5 µM) which was used as a positive control of the induction of apoptosis. After incubation cells were harvested by trypsinization, resuspended in fresh medium, stained with Annexin V and 7-AAD solution, subjected to 20 min incubation in the dark at room temperature, and analyzed by flow cytometry on Muse^®^ Cell Analyzer. Data were evaluated using Muse^®^ Analysis Software ver. 1.5 (Merck, Darmstadt, Germany).

#### 2.7.4. The Level of ROS

The Muse^®^ Oxidative Stress Kit (Merck, Darmstadt, Germany) was used according to the manufacturer’s protocol for quantitative measurement of superoxide radicals. The detection is based on the reaction of dihydroethidium (DHE) with superoxide radicals, which generates fluorophores with a high affinity to DNA. Briefly, cells (3 × 10^5^ per well) were seeded in the 6-well plates, pre-incubated for 24 h, and further grown for 24 h in the presence of the tested compounds. Subsequently, cells were collected, resuspended in 1X Assay Buffer, mixed with Muse^®^ Oxidative Stress Reagent working solution, and finally incubated for 30 min at 37 °C. Cells were analyzed by flow cytometry on Muse^®^ Cell Analyzer, and data were evaluated using Muse^®^ Analysis Software ver. 1.5 (Merck, Darmstadt, Germany).

### 2.8. In Vivo Evaluation of the Anticancer Potential of XN, PEITC, and Their Combination

All experiments were approved by the Local Ethics Committee for Animal Research in Poznan, Poland (date of approval: 05/04/2019; decision 18/2019), and carried out according to the ethical guidelines from Directive 2010/63/EU of the European Parliament on the protection of animals used for scientific purposes and Polish regulations.

For this study, thirty-two male BALB c/Nude mice were purchased from Charles River (Sulzfeld, Germany). Mice were maintained in a specific pathogen-free animal facility at the Center for Advanced Technology, Adam Mickiewicz University (Poznan, Poland) and housed under controlled temperature and humidity and 12 h light/dark cycle with ad libitum access to water and food.

#### 2.8.1. Mice Xenograft Model

Human pancreatic carcinoma PSN-1 cells were grown as indicated in Section 2.2. Cells were transfected according to our well-established protocol. Briefly, cells were transfected with constitutive reporter vector pGL4.51 (l*uc2*/CMV/Neo) (Promega, Madison, WI, USA) using ViaFect (Promega, Madison, WI, USA) as a transfection reagent. ViaFect Transfection Reagent:DNA complex (ratio 4:1) was added to the cells in a serum-free medium. After 48 h of incubation, the medium was removed, and the cells were incubated for two weeks in a selective medium (RPMI-1640 medium with 10% FBS, without penicillin/streptomycin) with Geneticin (G418, ThermoFisher, Waltham, MA, USA) as a selective agent. G418 was used at a concentration of 600 µg/mL.

The PSN-1-Luc cells (2 × 10^6^ cells/0.1 mL) were subcutaneously inoculated into the right flank of the mice (12–14 weeks old) under isoflurane anesthesia using a low-flow anesthesia system (Kent Scientific Corporation, Torrington, CT, USA). The cells were suspended in cold PBS and mixed with Matrigel (30%) (Corning Life Science, Tewksbury, MA, USA). Once the tumor became palpable 7–15 days after inoculation, the tumor size was measured using a digital caliper and calculated by the formula of an ellipsoid (length × width^2^) × 0.5 for tumor volume. On day 4, bioluminescence imaging (BLI) was done to evaluate the implantation success rate. The bioluminescence imaging was performed using PhotonImager (BiospaceLab, Nesles-la-Vallée, France) system. Moreover, BLI was performed on days 4, 11, 18, and 21 to investigate the optimal time for starting treatment). In this model, we observed the bioluminescent signals stabilization on day 21 post-implantation. Thus, according to these results, the treatment started on day 22. The tumor growth was also monitored by tumor size measurements using a caliper.

#### 2.8.2. Treatment Protocol

The tested compounds were dissolved in a vehicle consisting of ethanol 96%, PEG-400 (Sigma Aldrich, St Louis, MI, USA), and sterile sodium chloride 0.9%. Twenty-one days after implantation, mice were assigned into four groups: (1) Vehicle control group (*n* = 6); (2) XN-treated group (*n* = 7); (3) PEITC-treated group (*n* = 6); and (4) XN+PEITC-treated group (*n* = 7). The vehicle, XN (40 mg/kg b.w.), PEITC (15 mg/kg b.w.) and its combination were injected intraperitoneally (i.p.) into mice on day 22, 25, and 29 (days post-implantation). The effect of therapy was evaluated by BLI on days 26, 31, and 34, while tumor size was measured by caliper on days 20, 22, 25, 27, 29, 32, and 35. On day 35 after implantation, mice were sacrificed, and the tumor tissues were harvested, weighed, and measured.

### 2.9. Statistical Analysis

GraphPad Prism 9 (GraphPad Software, San Diego, CA, USA) was used to analyze the data. To assess the significance of the differences in the evaluated parameters one-way ANOVA with Dunnett’s post-hoc test was performed with the significance level of *p* < 0.05.

## 3. Results

### 3.1. Cell Viability after Treatment with Single Phytochemicals and Their Mixtures

**Figure 2:**
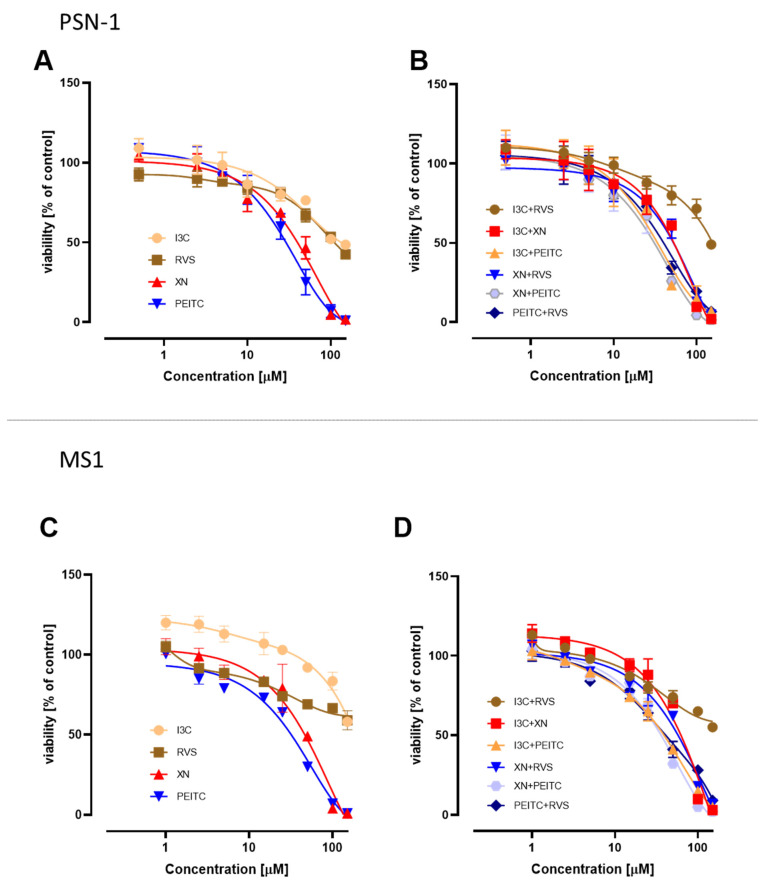
Cell viability changes in the PSN-1 cells (panel **A**,**B**) and MS1 (panel **C**,**D**) incubated for 24 h with XN, PEITC, RVS, I3C, and their mixtures. The values were estimated in comparison with vehicle control (100% viability). The results are presented as the mean ± SD calculated from three independent experiments.

The effect of XN, RVS, PEITC, I3C, and their combinations on the viability of PSN-1 (Figure 2A,B) and MS1 (Figure 2C,D) cells was evaluated by the MTT assay. In the case of single phytochemicals, we noticed the highest cytotoxicity in both cell lines for PEITC. From all the tested combinations XN+PEITC possessed the highest cytotoxic effect in both non-cancerous and cancer cells but the effect in cancer cells was more pronounced. However, the applied combinations of chemicals did not lead to the enhancement of cytotoxic effects in comparison to single compounds. The IC_50_ values are presented in Table 2.

### 3.2. The Canonical and Non-Canonical Mechanism of Nrf2 Activation

**Figure 3:**
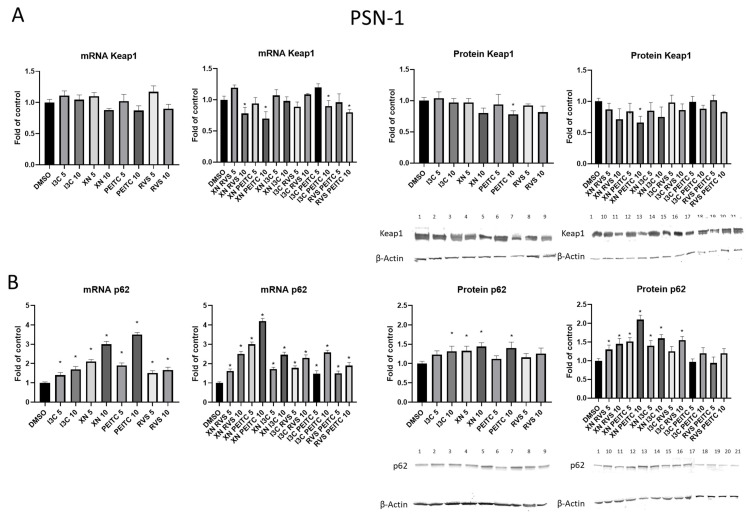
The effect of XN, PEITC, RVS, I3C, and their mixtures on the expression of two key components of canonical and non-canonical activation pathways—Keap1 and p62 in PSN-1 cells. Panel (**A**) presents the results of XN, PEITC, RVS, I3C, and their mixtures on transcript and protein level with representative immunoblots for Keap1 in PSN-1 cells. Panel (**B**) presents the results of XN, PEITC, RVS, I3C, and their mixtures on transcript and protein level with representative immunoblots for p62 in PSN-1 cells. Results of the R-T PCR and Western blot analysis are calculated as mRNA and protein level in comparison with vehicle control, respectively. The values are shown as the mean ± SD calculated from three independent experiments (a fold of control). Significance of changes was determined by one-way ANOVA, post-hoc Dunnett’s test (* *p* < 0.05). Lane 1, DMSO; lane 2, I3C 5 µM; lane 3, I3C 10 µM; lane 4, XN 5 µM; lane 5, XN 10 µM; lane 6, PEITC 5 µM; lane 7, PEITC 10 µM; lane 8, RVS 5 µM; lane 9 RVS 10 µM; lane 10, XN + RVS 5 µM; lane 11, XN + RVS 10 µM; lane 12, XN + PEITC 5 µM; lane 13, XN + PEITC 10 µM; lane 14, XN + I3C 5 µM; lane 15, XN + I3C 10 µM; lane 16, I3C + RVS 5 µM; lane 17, I3C + RVS 10 µM; lane 18, I3C + PEITC 5 µM; lane 19, I3C + PEITC 10 µM; lane 20, RVS + PEITC 5 µM; lane 21, RVS + PEITC 10 µM.

To investigate the exact mechanism of the Nrf2 activation, we first decided to measure the effect of the tested compounds on the expression of two key components of both canonical and non-canonical activation pathways—Keap1 and p62, respectively. The mRNA level of *Keap1* was decreased in PSN-1 cells by the treatment with combinations: XN+RVS, XN+PEITC, I3C+PEITC, and RVS+PEITC at the higher concentration (Figure 3A). Moreover, we observed reduced Keap1 protein expression in the cells after treatment with PEITC, XN+PEITC at the concentration of 10 μM. On the other hand, the expression of the *p62* gene in PSN-1 cell line was increased as a result of treatment with all the compounds and their combinations. A similar effect was observed at the protein level (Figure 3B). From all of the tested compounds XN at both concentrations, I3C and PEITC at the concentration 10 μM as well as most of the combinations: XN+RVS, XN+PEITC, XN+I3C at both concentrations, and I3C+RVS (10 μM) significantly augmented the p62 protein level.

**Figure 4:**
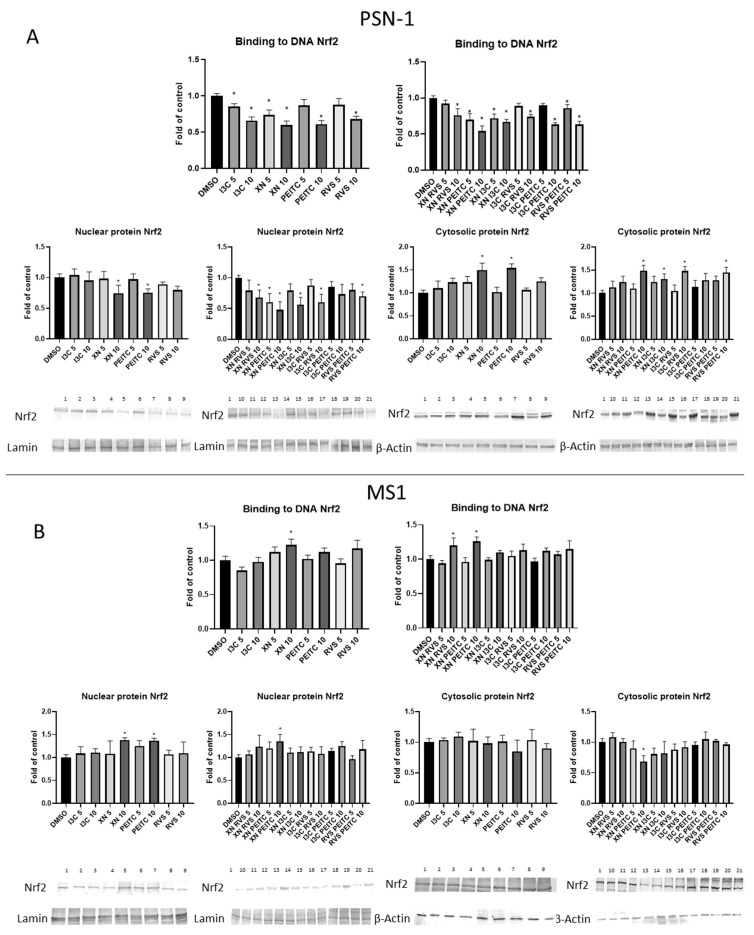
The effect of XN, PEITC, RVS, I3C, and their mixtures on the activation of the Nrf2 signaling pathway in PSN-1 and MS1 cells. Panel (**A**) presents the activation of Nrf2 estimated in terms of the amount of Nrf2 contained in the DNA-binding complexes extracted from the nuclear fraction. Exemplary immunoblots for the analysis of the cytosolic and the nuclear level of Nrf2 protein in PSN-1 cells are shown. Panel (**B**) presents the activation of Nrf2 estimated in terms of the amount of Nrf2 contained in the DNA-binding complexes extracted from the nuclear fraction. Exemplary immunoblots for the analysis of the cytosolic and the nuclear level of Nrf2 protein in MS1 cells are shown. Results of the Western blot analysis are calculated as protein level in comparison with vehicle control. The values are shown as the mean ± SD calculated from three independent experiments (a fold of control). Significance of changes was determined by one-way ANOVA, post-hoc Dunnett’s test (* *p* < 0.05). Lane 1, DMSO; lane 2, I3C 5 µM; lane 3, I3C 10 µM; lane 4, XN 5 µM; lane 5, XN 10 µM; lane 6, PEITC 5 µM; lane 7, PEITC 10 µM; lane 8, RVS 5 µM; lane 9 RVS 10 µM; lane 10, XN + RVS 5 µM; lane 11, XN + RVS 10 µM; lane 12, XN + PEITC 5 µM; lane 13, XN + PEITC 10 µM; lane 14, XN + I3C 5 µM; lane 15, XN + I3C 10 µM; lane 16, I3C + RVS 5 µM; lane 17, I3C + RVS 10 µM; lane 18, I3C + PEITC 5 µM; lane 19, I3C + PEITC 10 µM; lane 20, RVS + PEITC 5 µM; lane 21, RVS + PEITC 10 µM.

Next, we examined how the changes in the expression of regulatory proteins affected the activation of the Nrf2 signaling pathway. We performed the Nrf2-ARE binding assay, and we could observe the significant decrease in the level of binding of active nuclear Nrf2 to oligonucleotides containing its consensus sequence after treatment with all the analyzed compounds and their combinations in pancreatic cancer cells (Figure 4A). Furthermore, we noticed a remarkable decrease at the nuclear protein level and an increase at the cytosolic Nrf2 protein level as the effect caused by most of the tested combinations of the compounds in PSN-1 cells (Figure 4A). In the non-cancerous MS1 cell line the pattern of changes was opposite. We noticed increased binding to the ARE sequence as a result of single compound XN, and mixtures XN+RVS and XN+PEITC at the concentration 10 μM. In addition, we detected elevated levels of nuclear protein for XN, PEITC, and their combination and also the diminished level of the cytosolic protein after treatment with XN+PEITC at the higher concentration (Figure 4B).

**Figure 5:**
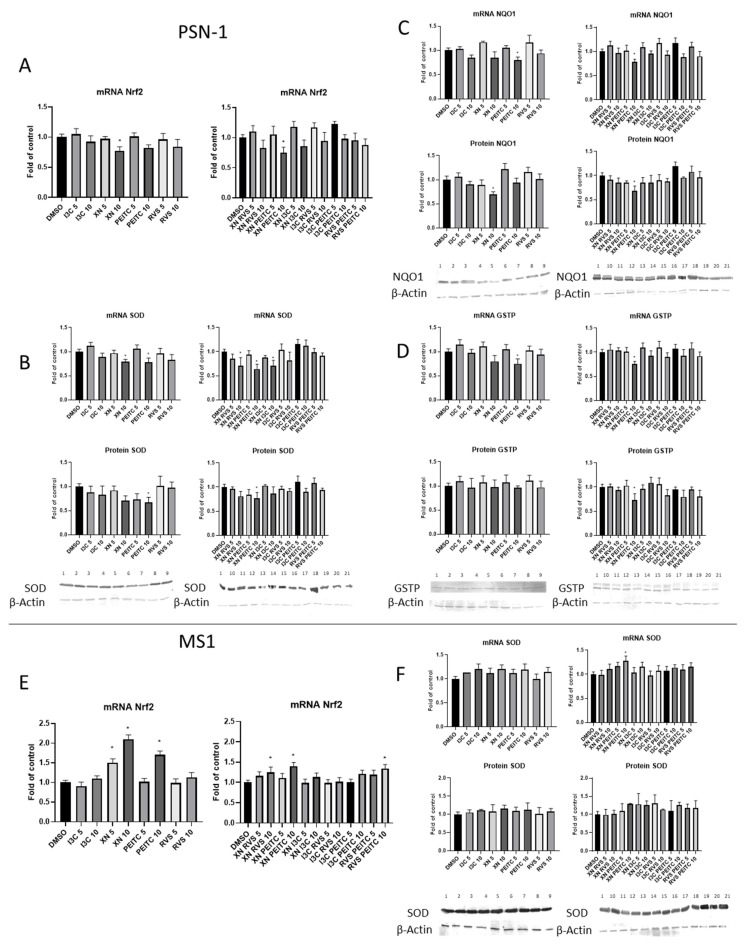
The effect of XN, PEITC, RVS, I3C, and their mixtures on the expression of Nrf2 and its target genes in PSN-1 and MS1 cells. The expression of Nrf2 (PSN-1—panel **A**; MS1—panel **E**), SOD (PSN-1—panel **B**; MS1—panel **F**), NQO1 (PSN-1—panel **C**) and GSTP (PSN-1—panel **D**) was estimated as mRNA level in comparison with control cells. Exemplary immunoblots for the analysis of protein level of SOD (PSN-1—panel **B**; MS1—panel **F**), NQO1 (PSN-1—panel **C**), and GSTP (PSN-1—panel **D**) are presented. Results of the Western blot analysis are calculated as protein level in comparison with vehicle control. The values are shown as the mean ± SD calculated from three independent experiments (a fold of control). Significance of changes was determined by one-way ANOVA, post-hoc Dunnett’s test (* *p* < 0.05). Lane 1, DMSO; lane 2, I3C 5 µM; lane 3, I3C 10 µM; lane 4, XN 5 µM; lane 5, XN 10 µM; lane 6, PEITC 5 µM; lane 7, PEITC 10 µM; lane 8, RVS 5 µM; lane 9 RVS 10 µM; lane 10, XN + RVS 5 µM; lane 11, XN + RVS 10 µM; lane 12, XN + PEITC 5 µM; lane 13, XN + PEITC 10 µM; lane 14, XN + I3C 5 µM; lane 15, XN + I3C 10 µM; lane 16, I3C + RVS 5 µM; lane 17, I3C + RVS 10 µM; lane 18, I3C + PEITC 5 µM; lane 19, I3C + PEITC 10 µM; lane 20, RVS + PEITC 5 µM; lane 21, RVS + PEITC 10 µM.

To assess the consequences of the inhibited activity of the Nrf2 pathway, we measured the expression of the genes regulated by this transcription factor—namely *SOD, NQO1*, and *GSTP*. The levels of their transcripts and proteins were determined. In the case of PSN-1, cells the expression of the above-mentioned parameters was mainly diminished (Figure 5A–D). The mRNA level of *SOD* was significantly decreased by single XN and PEITC, and three of the tested combinations—XN+RVS, XN+PEITC, and XN+I3C—at the concentration of 10 μM. We confirmed these differences at the protein level for PEITC (10 μM) and XN+PEITC (10 μM) (Figure 5B). The mRNA and protein level of NQO1 was reduced as a result of the treatment with combination of XN with PEITC at a higher dosage (Figure 5C). We noticed a significant change for GSTP at the mRNA and protein level for the XN+PEITC at a higher dose (Figure 5D).

In comparison to the cancer cells, in the non-cancerous MS1 cell line, the level of *Nrf2* mRNA was increased by XN (5 μM, 10 μM) and PEITC (10 μM) as well as by three combinations XN+RVS, XN+PEITC, and RVS+PEITC at the concentration 10 μM (Figure 5E). Interestingly, we did not detect significant consequences of these changes in the protein level, but we noticed an increase in the transcript level of *SOD* caused by XN+PEITC 10 μM (Figure 5F).

### 3.3. Interaction of Nrf2 with NF-κB Signaling Pathway

**Figure 6:**
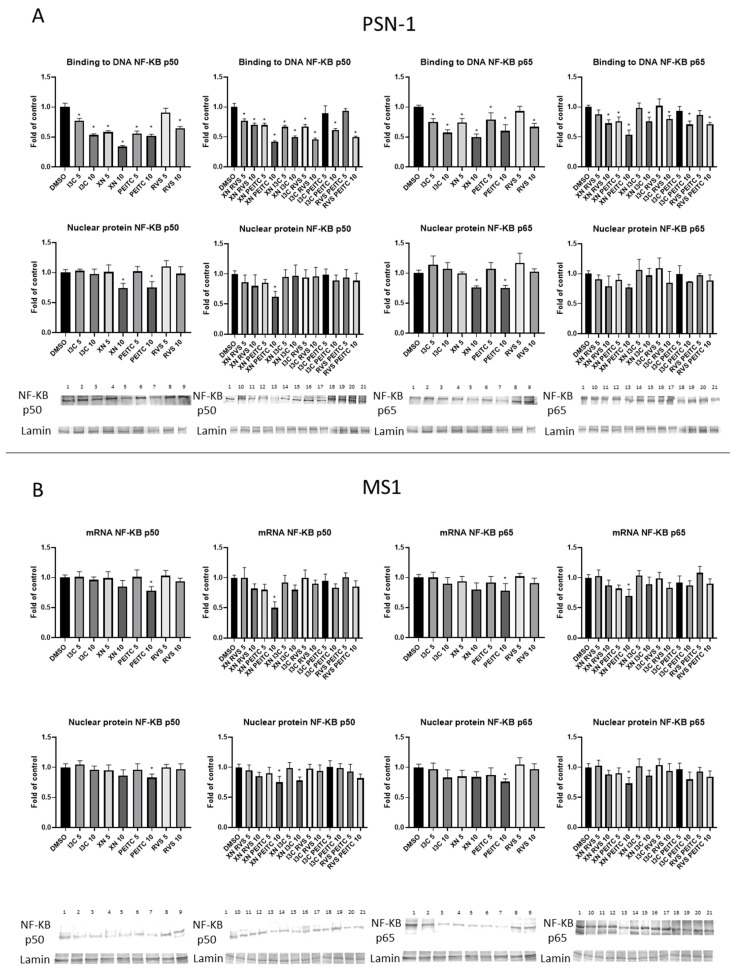
Effect of XN, PEITC, RVS, I3C, and their mixtures on the activation of NF-κB signaling pathway in PSN-1 and MS1 cells. Panel (**A**) presents the activation of NF-κB estimated in terms of the amount of NF-κB p50 and NF-κB p65 contained in the DNA-binding complexes extracted from the nuclear fraction in PSN-1 cells. Exemplary immunoblots for the analysis of the nuclear level of NF-κB p50 and NF-κB p65 protein in PSN-1 cells are shown. Panel (**B**) presents the transcript and protein level with representative immunoblots for NF-κB p50 and NF-κB p65 in MS1 cells. Results of the R-T PCR and Western blot analysis are calculated as mRNA and protein level in comparison with vehicle control, respectively. The values are shown as the mean ± SD calculated from three independent experiments (a fold of control). Significance of changes was determined by one-way ANOVA, post-hoc Dunnett’s test (* *p* < 0.05). Lane 1, DMSO; lane 2, I3C 5 µM; lane 3, I3C 10 µM; lane 4, XN 5 µM; lane 5, XN 10 µM; lane 6, PEITC 5 µM; lane 7, PEITC 10 µM; lane 8, RVS 5 µM; lane 9 RVS 10 µM; lane 10, XN + RVS 5 µM; lane 11, XN + RVS 10 µM; lane 12, XN + PEITC 5 µM; lane 13, XN + PEITC 10 µM; lane 14, XN + I3C 5 µM; lane 15, XN + I3C 10 µM; lane 16, I3C + RVS 5 µM; lane 17, I3C + RVS 10 µM; lane 18, I3C + PEITC 5 µM; lane 19, I3C + PEITC 10 µM; lane 20, RVS + PEITC 5 µM; lane 21, RVS + PEITC 10 µM.

**Figure 7:**
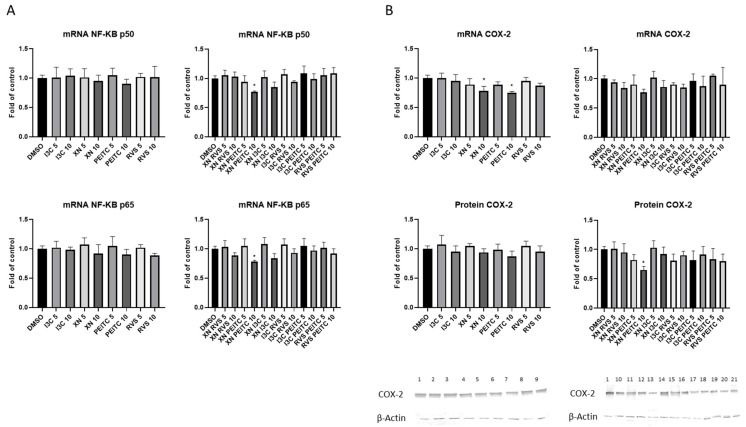
Effect of XN, PEITC, RVS, I3C, and their mixtures on the expression of NF-κB and its target gene—*COX-2* in PSN-1 cells. The expression of *NF-κB p50* and *NF-κB p65* (panel **A**), and *COX-2* (panel **B**) was estimated as mRNA level in comparison with control cells. Exemplary immunoblots for the analysis of protein level of COX-2 (panel **B**) are presented. Results of the R-T PCR and Western blot analysis are calculated as mRNA and protein level in comparison with vehicle control, respectively. The values are shown as the mean ± SD calculated from three independent experiments (a fold of control). Significance of changes was determined by one-way ANOVA, post-hoc Dunnett’s test (* *p* < 0.05). Lane 1, DMSO; lane 2, I3C 5 µM; lane 3, I3C 10 µM; lane 4, XN 5 µM; lane 5, XN 10 µM; lane 6, PEITC 5 µM; lane 7, PEITC 10 µM; lane 8, RVS 5 µM; lane 9 RVS 10 µM; lane 10, XN + RVS 5 µM; lane 11, XN + RVS 10 µM; lane 12, XN + PEITC 5 µM; lane 13, XN + PEITC 10 µM; lane 14, XN + I3C 5 µM; lane 15, XN + I3C 10 µM; lane 16, I3C + RVS 5 µM; lane 17, I3C + RVS 10 µM; lane 18, I3C + PEITC 5 µM; lane 19, I3C + PEITC 10 µM; lane 20, RVS + PEITC 5 µM; lane 21, RVS + PEITC 10 µM.

To examine the activation of NF-κB, we performed the NF-κB binding assay. The significant inhibition of NF-κB p50 and p65 binding was noticed after treatment with all analyzed compounds and their combinations (Figure 6A) in PSN-1 cells. Moreover, the NF-κB p50 nuclear protein level measured by Western blot was diminished after treatment with XN+PEITC 10 μM and single components of this combination (Figure 6A). XN and PEITC alone at higher concentration also decreased the protein level of NF-κB p65 subunit. We observed significant differences in the transcript level of NF-κB p50 and p65 (Figure 7A) for XN+PEITC 10 μM. Since *COX-2* is one of the NF-κB target genes as the result of diminished activation of NF-κB by XN+PEITC 10 μM, a reduced level of COX-2 protein was observed (Figure 7B).

In the non-cancerous MS1 cells, we also confirmed inhibition of the NF-κB signaling pathway. We noticed significant decrease in the mRNA and protein level of NF-κB p50 and NF-κB p65 as a result of PEITC and its combination with XN (Figure 6B).

Glycogen synthase kinase-3β (GSK3β) has been shown to attenuate the transcriptional activity of Nrf2 [28] and has been reported to be necessary for the total transcriptional activity of NF-κB, demonstrating that GSK3β selectively supports the expression of a subset of genes activated by NF-κB-dependent proliferative signals [29]. These observations indicate the existence of a crosstalk between Nrf2 and NF-κB pathways.

**Figure 8:**
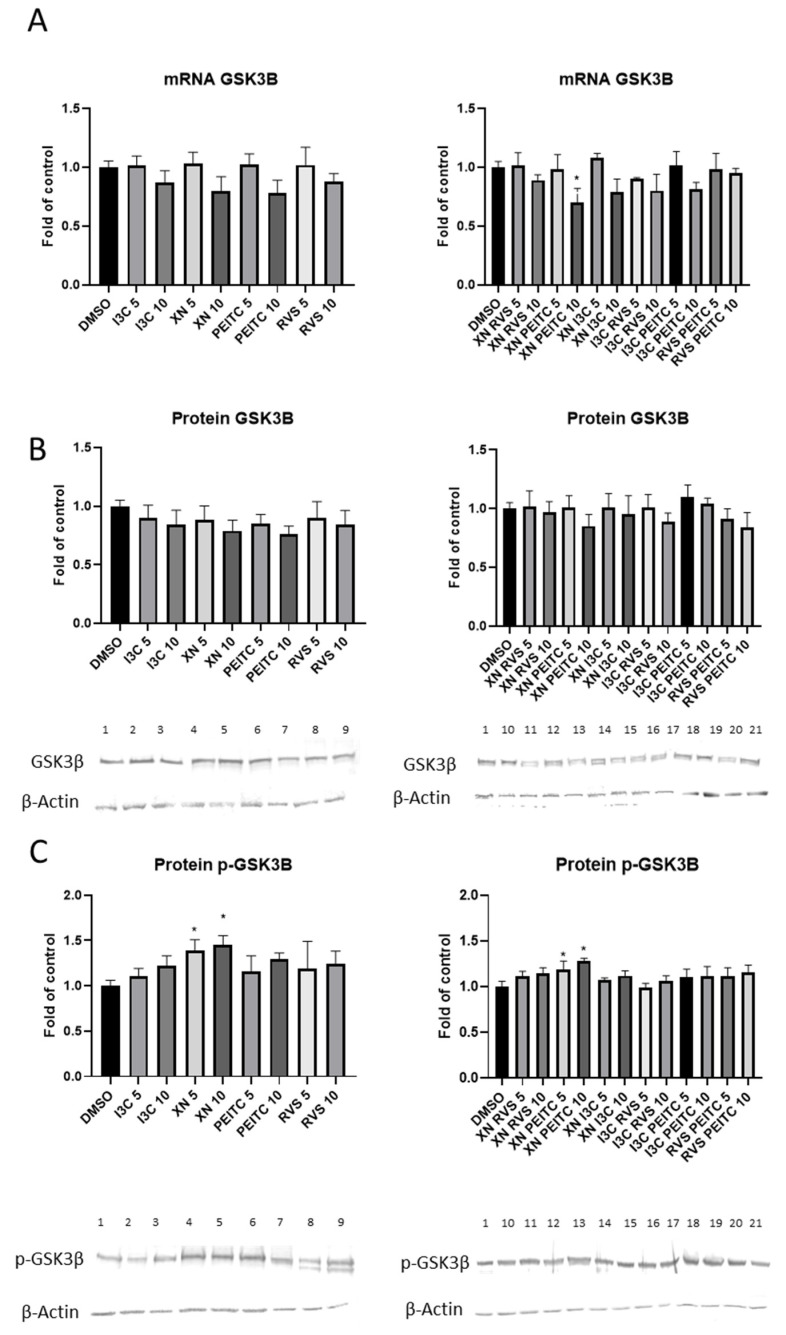
Activation of GSK3β and its phosphorylated form by XN, PEITC, RVS, I3C, and their mixtures in PSN-1 cells. Panel (**A**) presents the relative changes in the GSK3β expression estimated as mRNA level in comparison with control cells. Results of the Western blot analysis of GSK3β (panel **B**) and p-GSK3β (panel **C**) are calculated as protein level in comparison with vehicle control. Exemplary immunoblots for the analysis of protein level of GSK3β and p-GSK3β are presented. The values are shown as the mean ± SD calculated from three independent experiments (a fold of control). Significance of changes was determined by one-way ANOVA, post-hoc Dunnett’s test (* *p* < 0.05). Lane 1, DMSO; lane 2, I3C 5 µM; lane 3, I3C 10 µM; lane 4, XN 5 µM; lane 5, XN 10 µM; lane 6, PEITC 5 µM; lane 7, PEITC 10 µM; lane 8, RVS 5 µM; lane 9 RVS 10 µM; lane 10, XN + RVS 5 µM; lane 11, XN + RVS 10 µM; lane 12, XN + PEITC 5 µM; lane 13, XN + PEITC 10 µM; lane 14, XN + I3C 5 µM; lane 15, XN + I3C 10 µM; lane 16, I3C + RVS 5 µM; lane 17, I3C + RVS 10 µM; lane 18, I3C + PEITC 5 µM; lane 19, I3C + PEITC 10 µM; lane 20, RVS + PEITC 5 µM; lane 21, RVS + PEITC 10 µM.

We sought to study the activation of GSK3β and its phosphorylated form in PSN-1 cells. The combination XN+PEITC 10 μM significantly reduced GSK3β transcript level (Figure 8A). The same combination in both doses increased the phosphorylated GSK3β protein level (Figure 8C), however, it did not significantly affect its unphosphorylated form (Figure 8B). In non-cancerous MS1 cells we did not observe significant changes in phosphorylated GSK3β protein level after treatment with phytochemicals (data not shown).

### 3.4. Multiplex Analysis of the Level of Proteins Regulating Several Signaling Pathways

**Figure 9:**
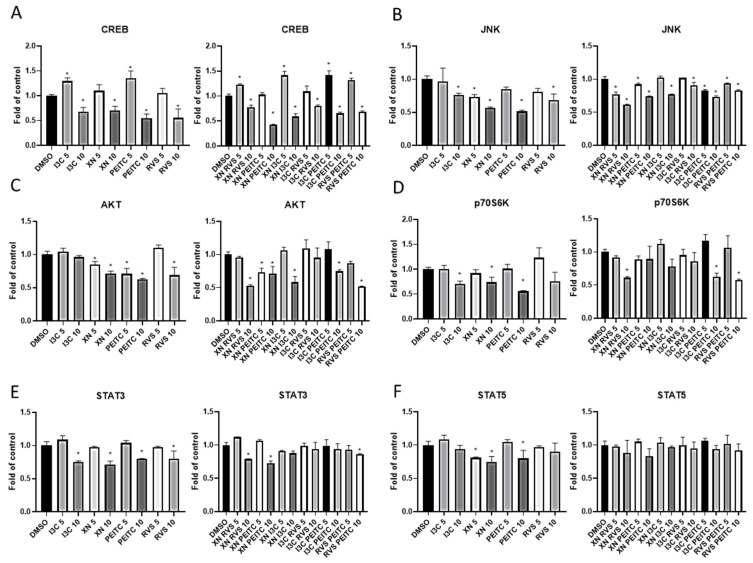
Effect of XN, PEITC, RVS, I3C, and their mixtures on the regulation of protein controlling several signaling pathways measured by bead-based multiplex immunoassay in PSN-1. Results are prepared based on the cytosolic fraction of proteins and are shown in comparison to vehicle control. Panel (**A**) presents the relative changes in the protein level of CREB, panel (**B**) presents the relative changes in the protein level of JNK, panel (**C**) presents the relative changes in the protein level of AKT, panel (**D**) presents the relative changes in the protein level of p70S6K, panel (**E**) presents the relative changes in the protein level of STAT3, panel (**F**) presents the relative changes in the protein level of STAT5. The values are shown as the mean ± SD calculated from three independent experiments (a fold of control). Significance of changes was determined by one-way ANOVA, post-hoc Dunnett’s test (* *p* < 0.05).

It is well established that changes in one signaling pathway can result in a significant interruption of cellular homeostasis. In order to look into possible interactions of the tested compounds and their mixes with signaling pathways responsible for maintaining homeostasis and cell survival, other than Nrf2 and NF-κB, we decided to apply the bead-based multiplex immunoassay in PSN-1 cells. As shown in Figure 8, all of the compounds and their combinations at higher concentration reduced the level of cAMP response element-binding protein (CBREB), known to be highly overexpressed in pancreatic cancers (Figure 9A). However, this effect appeared to be concentration-dependent, we observed the opposite results at the lower concentration of 5 μM. Moreover, the significant reduction in JNK level (Figure 9B) after treatment with XN, RVS, PEITC, and I3C—and all of the tested combinations—was noticed. The effect was dose-dependent in the case of phytochemicals and their mixtures. Furthermore, most of the analyzed phytochemicals diminished the levels of other kinases from the MAPK family, AKT (Figure 9C; Figure S2) and p70S6K (Figure 9D). The most pronounced effect was confirmed for XN and PEITC 10 μM and their combinations with other phytochemicals. The Signal Transducer and Activator of Transcription (STAT) 3 and 5 proteins’ roles in cancer are largely context-dependent as they can both act as oncogenes and tumor suppressors. XN and PEITC at the higher concentration reduced the level of both STAT3 and STAT5, but their combination was effective only in the case of STAT3 (Figure 9E,F).

These results indicate that analyzed phytochemicals affected not only Nrf2 and NF-κB signaling pathways, but also could influence the level of proteins regulating signaling pathways responsible for apoptosis, cell cycle distribution, cell migration, and protein synthesis in the pancreatic cancer cell line.

### 3.5. Analysis of Apoptosis and the Level of ROS

**Figure 10:**
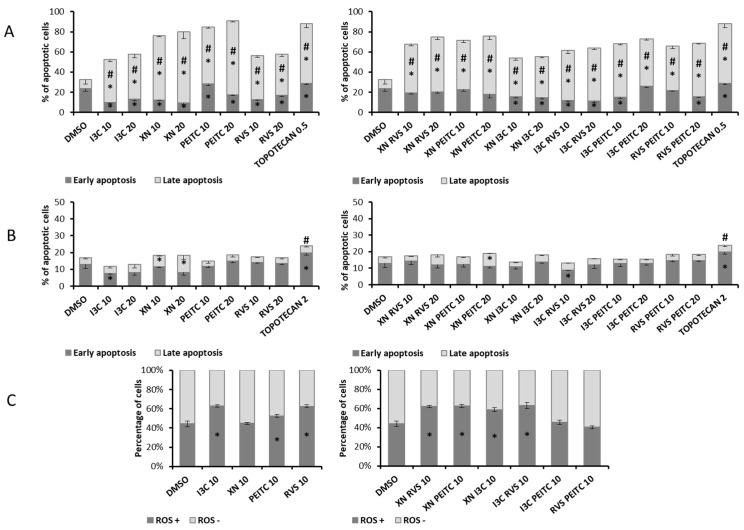
Effect of XN, PEITC, RVS, I3C, and their mixtures on apoptosis PSN-1 and MS1 cells and on the level of ROS in PSN-1 cells. Panel (**A**): the percentage of cells in the early and late stage of the apoptosis in PSN-1 cells; panel (**B**): the percentage of cells in the early and late stage of the apoptosis in MS1 cells; both evaluated by the flow cytometry-based on fluorescence signal from Annexin V bound to phosphatidylserine externalized in apoptotic cells and a dead cell marker 7-AAD. Panel (**C**): the percentage of cells undergoing oxidative stress based on intracellular detection of superoxide radicals—reactive oxygen species positive (ROS (+)) and negative (ROS (−)). Results were evaluated from three separate experiments (mean ± SD) and determined by one-way ANOVA, post-hoc Dunnett’s test. Asterisk (*) indicates statistically significant differences from the control group, while hashes (#) indicate statistically significant changes in the percentage of total apoptotic cells, *p* < 0.05.

To evaluate apoptosis in pancreatic cancer PSN-1 cells and endothelial pancreatic MS1 cells we used The Muse^®^ Annexin V and Dead Cell Kit. PSN-1 are extensively proliferating pancreatic adenocarcinoma cancer cells with a high basal level of early apoptosis, but all the tested chemicals were able to meaningfully increase the population of late apoptotic cells. Both XN and PEITC were the most active compounds (77–91% of total apoptosis), while three mixtures: XN + RVS, XN + PEITC, I3C + PEITC were able to induce the total apoptosis exceeding 70% (Figure 10A). In the case of MS1 cell line, we observed the inhibition of apoptosis, but XN and its combination with PEITC did increase the level of cells in the late apoptosis stage (Figure 10B), although the differences were less remarkable than in PSN-1 cells.

Extensive apoptosis might be a result of oxidative stress induced in cells. Thus, we additionally analysed ROS level in the PSN-1 cell line. Approximately 44% of unstimulated cells were ROS positive, which is in line with intensive apoptosis detected in these cells. In addition, single compounds I3C, PEITC, RVS, and their combinations: XN + RVS, XN + PEITC, XN + I3C, and I3C + RVS elevated the population of ROS positive cells (Figure 10C). These results correspond with the decreased Nrf2 level and its inhibited activation in PSN-1 cells.

### 3.6. Analysis of Cell Proliferation and Cell Cycle Distribution

**Figure 11:**
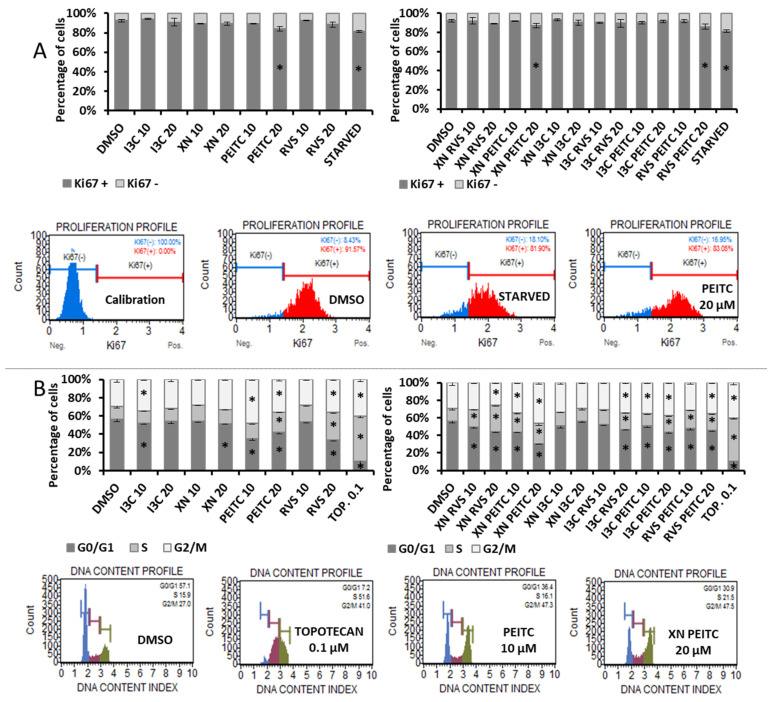
Effect of XN, PEITC, RVS, I3C, and their mixtures on cell proliferation and cell cycle distribution in PSN-1 cells. Panel (**A**): the percentage of proliferating (Ki67 (+)) and non-proliferating (Ki67 (−)) cells were analyzed by flow cytometry. As a reference to antiproliferative conditions, starved cells (none FBS medium) were used. Panel (**B**): the percentage of cells in the G1/G0, S and G2/M phase analyzed by the flow cytometry after staining with propidium iodide and RNase A. Topotecan 0.1 µM (TOP. 0.1) was used as a positive control. Exemplary plots are presented. Results were calculated from three separate experiments (mean ± SD) and determined by one-way ANOVA, post-hoc Dunnett’s test. Asterisk (*) indicates statistically significant differences from the control group, *p* < 0.05.

The proliferation of PSN-1 cells was evaluated in terms of the expression of Ki67 protein—the marker of proliferating cells which is absent only in the G0 phase of cell cycle. Among single compounds, we observed a slight reduction of proliferation rate as a result of PEITC 20 μM treatment (Figure 11A), where the percentage of non-proliferating cells was doubled, as for starved cells. The combinations of PEITC with XN or with RVS gave similar results.

Moreover, we assessed the effect of the compounds on cell cycle distribution in PSN-1 cells. The application of all single chemicals affected the distribution of cells across the cell cycle to some extent, causing cell cycle arrest in the G2/M phase and a decrease in G0/G1 phase (Figure 11B). Similar changes were observed for mixes, the most pronounced effect was noticed in the case of the XN + PEITC combination. The enrichment of cells in G2/M and S phases was also observed for the topoisomerase inhibitor, topotecan, which was used as a positive control of cell cycle arrest.

### 3.7. Antitumor Efficacy of Xanthohumol, Phenethyl Isothiocyanate, and Their Combination in Human Pancreatic Tumor Xenograft Model

**Figure 12:**
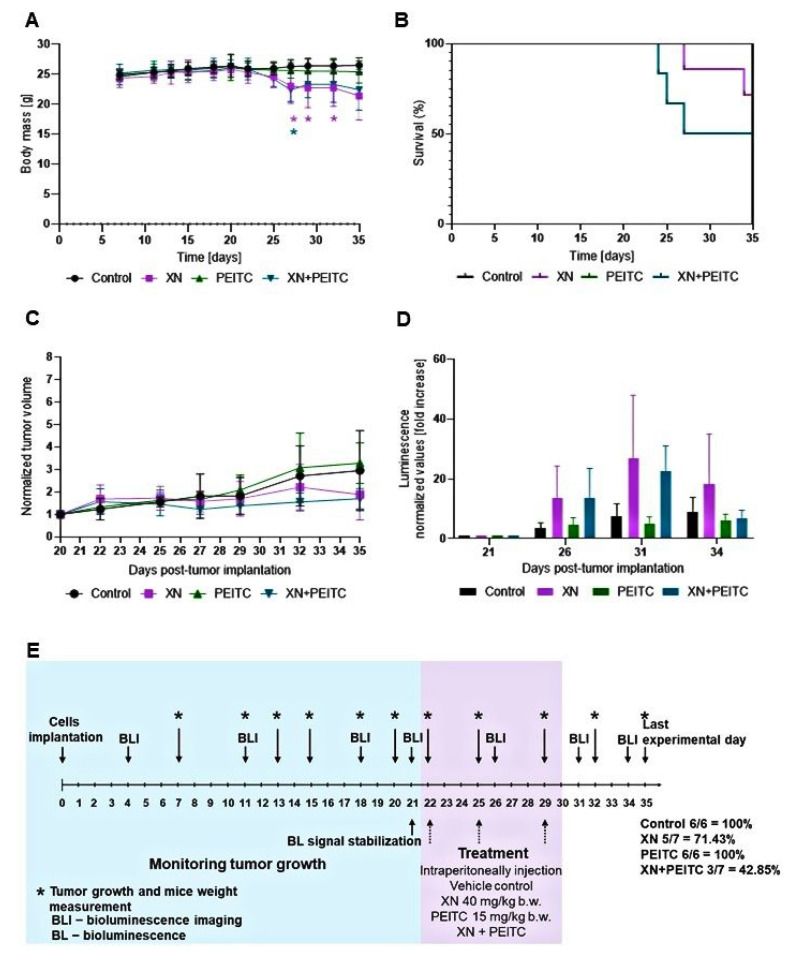
In vivo activity of XN, PEITC, and their combination. Panel (**A**) presents body weight changes in tumor-bearing mice; panel (**B**) presents the Kaplan–Meier survival curve of mice bearing PSN-1 xenografts; panel (**C**) presents the changes in tumor volume after treatment. Panel (**D**) presents the BLI results; panel (**E**) presents the schematic illustration of the study time frame. The bioluminescence imaging was performed using PhotonImager (BiospaceLab, Nesles-la-Vallée, France) system. The luminescence signal for each mouse was counted by selecting the region of interest (ROI) and quantifying as the total photons counts using the M3 software. The data are presented as means ± SD. Asterisk (*) indicates statistical significance, *p* < 0.05.

The schematic illustration of in vivo experiments is presented in Figure 12. The response to treatment for the two most active compounds—XN and PEITC, at a dose of 40 mg/kg b.w and 15 mg/kg b.w—was monitored by tumor volume measurement and BLI. The results from the performed experiment showed that the tested compounds and their combination did not significantly attenuate tumor growth. However, the tumor volume for the group treated with XN, and combination (XN with PEITC) was lower than the control group (Figure 12). The treatment of tumor-bearing mice with the combination of XN and PEITC at a dose of 40 mg/kg b.w and 15 mg/kg b.w, respectively, was associated with a decreased overall survival time (Figure 12). It should also be noted that some other possible factors might be responsible for this observation, e.g., progressive neoplastic disease, which could result in higher sensitivity to the tested xenobiotics.

**Figure 13:**
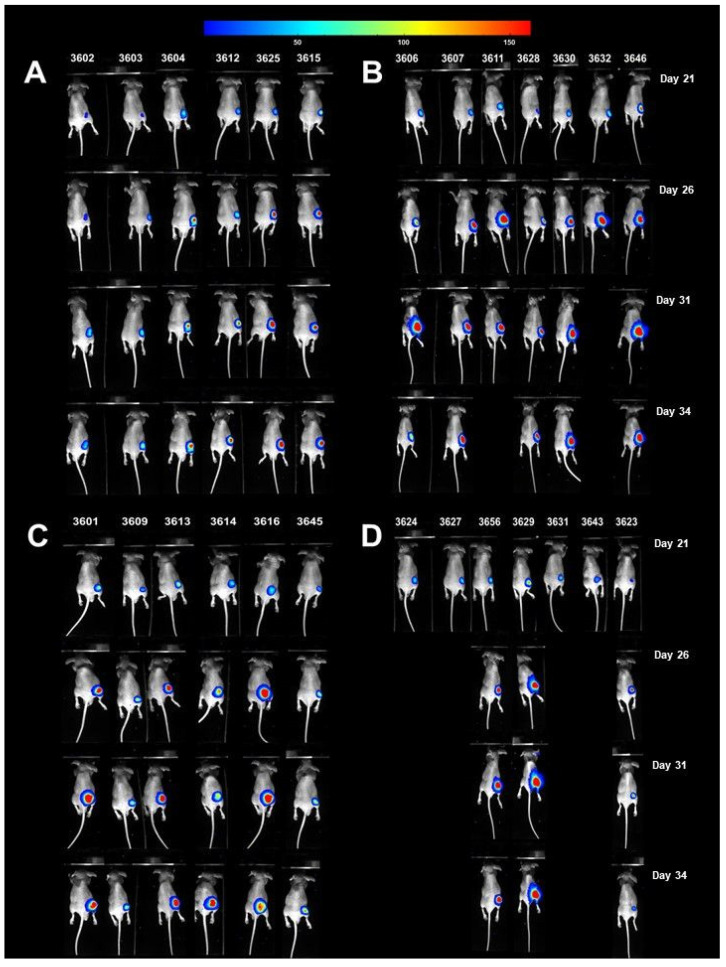
Bioluminescence imaging of tumor-bearing mice received the control vehicle (**A**), XN (**B**), PEITC (**C**), and a combination of XN with PEITC (**D**) on days 22, 25, and 29. The BLI was performed on days 21, 26, 31, and 34. Mice were injected intraperitoneally with 150 mg/kg b.w. D-Luciferin for evaluation of tumor burden and BLI was performed using the Photon Imager (BioSpace Lab).

It should be highlighted that some potentially toxic effects might be observed in the groups that received XN and the combination XN with PEITC. As indicated in Figure 12A, the changes in body weight of mice in group XN and XN+PEITC group were observed. The BLI studies showed that the tumor growth rate in the group that received PEITC alone did not change during the treatment period (Figure 12). The images from BLI experiments are presented in Figure 13.

**Figure 14:**
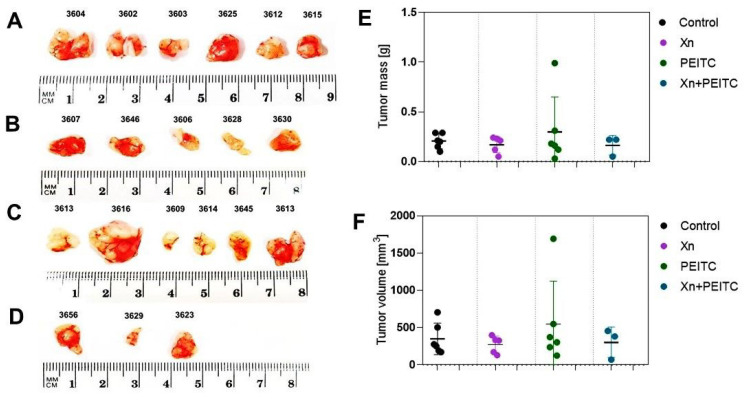
Ex vivo measurement of the tumor mass and volume. Panel (**A**) presents the tumor images of the control group; panel (**B**) tumors from the group received XN; panel (**C**) tumors from the group treated with PEITC; panel (**D**) presents the tumors group treated with a combination of XN and PEITC. Panel (**E**) presents tumor mass, while panel (**F**) tumor volume was measured ex vivo.

In general, tumor volume measurement by caliper showed the partial response in the group receiving xanthohumol alone (a reduction in the volume by ~36%), and the group receiving the combinatorial treatment (decrease by approximately 40%). However, these changes were not statistically significant. The ex vivo measurements of the tumor mass and tumor volume are presented in Figure 14. These data also show the partial response in the group treated with XN and the combination of XN and PEITC. However, further research is needed to evaluate the anticancer potential of the tested combination and to overcome the observed side effects.

## 4. Discussion

Pancreatic cancer is a significant problem in the scale of the entire society around the world. Statistical data indicate that its incidence by 2030 will increase significantly and pancreatic cancer will be one of the leaders in incidence and mortality [30]. Many studies have shown that proliferation, apoptosis, and cell cycle distribution are regulated by Nrf2 and NF-κB signaling pathways [31,32]. Recently, much attention has been paid to the interaction between these pathways to select potential therapeutic agents, targeting the above-mentioned transcription factors. The protective role of Nrf2 has been revealed in several models in which the oxidation of Keap1 by the electrophilic compounds (classical inducers) is essential for the Nrf2 activation and cell protection (canonical pathway). However, the classical inducers have unspecific activity since they can oxidize different serine residues in many proteins, producing side effects, which limits the use of these classic activators [5].

Under oxidative stress and in the presence of phytochemicals (electrophilic compounds), Nrf2 is activated and induces the expression of detoxifying and antioxidant enzymes, which are involved in normal cell protection. Alternatively, Nrf2 activation may occur by a non-canonical mechanism, where the disruption of the Keap1–Nrf2 complex is carried out by proteins such as p62, LC3, and DPP3 [33]. Accordingly, more specific compounds with fewer side effects must be found and the compounds and/or combinations acting through the non-canonical pathway seem to be an excellent option. Furthermore, targeting p62, or the enzymes that modify it, could be an advantageous strategy for treating diseases associated with the dysregulation of autophagy and prolonged activation of Nrf2 [33].

Our previous studies demonstrated the activation of the Nrf2 signaling pathway by combinations of phytochemicals to a higher extent than single compounds in pancreatic cancer cells PANC-1 [21] and Mia-Pa-Ca-2 cells [17]. These cell lines represent epithelioid carcinoma, but epidemiological data indicate that 90% of pancreatic cancers are adenocarcinomas [34].

In order to continue our research and precisely determine the effect of the combination of selected phytochemicals, in the next stage we chose the cancerous line PSN-1 representing adenocarcinoma and the non-cancerous cell line MS1 for comparison. The aim of this study was the estimation of the influence of phytochemicals combinations on the Nrf2 and NF-κB signaling pathways, and genes under their control, and to determine their interactions. Additionally, the impact of the most effective combination of phytochemicals on tumor growth was assessed using a mouse xenograft model.

The activation of Nrf2 involves the dissociation from Keap1 and the translocation of Nrf2 from the cytosol to nucleus and its binding to DNA and subsequent induction of antioxidant and cytoprotective genes. In the present study, we observed increased nuclear level of Nrf2 in non-cancerous pancreatic MS1 cells, especially after treatment with XN and PEITC and their combination. The opposite effect was shown in pancreatic cancer PSN-1 cells—XN, PEITC, and their combination in the higher doses diminished the nuclear level of Nrf2 and its binding to DNA. The reason for this discrepancy may lie in different basal levels of ROS in normal and tumor cells [35]. Several studies indicated that cancer cells produce higher levels of ROS than normal cells due to increased metabolic stress and proliferative capacity [36,37,38]. Moreover, normal cells are characterized by homeostasis in the regulation of signaling pathways, in contrast to cancer cells exhibiting deregulation of cell signaling, the mechanisms of this crosstalk may differ between these cell lines [39,40].

In the current study, we also observed an increase in the transcript and protein level of p62 accompanied by decreased mRNA and protein level of the Keap1 in PSN-1 cell line treated with the combinations of phytochemicals, especially XN and PEITC. Furthermore, a decreased activity of Nrf2 in PSN-1 cells correlated with the lower expression and protein levels of SOD, GSTP, and NQO1. We conclude that the modulation of the non-canonical and canonical activators of Nrf2 signaling pathway was not strong enough to affect the expression of Nrf2 dependent genes. Moreover, the reduced Keap1 level may result from secondary changes occurring during canonical and non-canonical activation of Nrf2, ubiquitination, epigenetic modifications, or the influence of kinases responsible for its phosphorylation [41,42]. Furthermore, since the Keap1 decrease and p62 increase were observed, we hypothesized possible modulation of the unknown mechanism of the translocation of Nrf2 to the nucleus. However, we strongly emphasize that further studies are necessary to elucidate the exact mechanism of this phenomenon.

Some data showed that the anti-cancer activity of phytochemicals involves the influence on many signaling pathways which control cell homeostasis. Interactions among Nrf2, NF-κB, and STAT3 signaling pathways, and their dysregulation, may play a key role in the development of cancer—including pancreatic cancer [22,43,44,45]—driven by the inflammation process. Signaling pathway NF-κB plays a crucial role in inflammatory response and production of pro-inflammatory molecules, including cytokines and enzymes such as COX-2 [46]. Inhibition of nuclear translocation and binding of NF-κB to DNA in cancer PSN-1 cells and non-cancerous MS1 cells exposed to XN, PEITC, and the combination of both confirms their anti-inflammatory potency. Moreover, this observation is in agreement with the results of our previous studies [17,21]. Among the pro-inflammatory enzymes, COX-2 is one of the most important regulators of angiogenesis, inflammation, and carcinogenesis [47,48]. The results of our current study suggest that decreased transcript and protein levels of COX-2 in PSN-1 cells are responsible for the anti-inflammatory properties of these compounds/phytochemicals.

Recent studies indicated that GSK-3β can regulate gene expression by activating or inhibiting some transcription factors—e.g., NF-κB and Nrf2 [49,50]. GSK-3β is a multifunctional kinase involved in various cellular functions, including differentiation, survival, protein synthesis, immune responses, and cell death [51]. In the present study, we showed that the tested compounds caused upregulation of p-GSK-3β in PSN-1 cells. These observations are in agreement with the results of the studies conducted by Gameiro’s and Wang’s groups [52,53]. Moreover pleiotropic function of GSK3β and its phosphorylation by many upstream kinases—e.g., Akt, PKC, Fyn—may suggest the potential crosstalk between Nrf2, NF-κB, and GSK3β. On the other hand, cancer originates from a disruption of homeostasis [39]. Since normal cells are characterized by homeostasis in the regulation of signaling pathways, in contrast to cancer cells exhibiting deregulation of cell signaling, the mechanisms of this crosstalk may differ between these cell lines [40]. However, this issue is still unclear which prompts us to undertake further research in this field.

Recently, it has been shown that Nrf2 opposes the NF-κB pathway by clashing with transcription co-activator cAMP response element (CREB) binding protein CBP [54]. CBP interacts with phosphorylated NF-κB p65, so the overexpression of p65 decreases the availability of CBP for Nrf2 connection [55]. The results from the study suggest that this mode of influence is highly plausible because we observed different modulation of CREB depending on phytochemicals dose in pancreatic cancer cells. Additionally, we demonstrated that transcription factors STAT3 and STAT5 were diminished by phytochemicals and their combinations. The decreased level of STATs corresponded with the inhibition of NF-κB and the cell cycle arrest. To further explain the mechanism of cell death induced by phytochemicals and combinations, their impact on cell cycle distribution and apoptosis was estimated. Our previous study indicated that the combination of XN and PEITC exerted antiproliferative effects through G0/G1 arrest [17], but our current results showed that this combination inhibited proliferation by the arrest in the G2/M phase. A variety of cellular activities—including metabolism, growth, and death—are regulated and modulated by the redox status of the environment. A biphasic effect has been demonstrated on cellular proliferation with ROS, in which low levels induce growth but higher concentrations induce apoptosis or necrosis. Much of the work examining the signal transduction by ROS has been performed using different doses. Although the use of higher ROS doses has allowed the identification of important signal transduction pathways, these pathways may be activated by cells only in association with ROS-induced apoptosis and necrosis, and may not utilize the same pathways activated by lower doses of ROS associated with increased cell growth [56]. Recent data has shown that low levels of exogenous H_2_O_2_/ROS upregulate intracellular glutathione and activate the DNA binding activity toward antioxidant response element [56,57]. Nrf2 activates the expression of antioxidant and detoxifying enzymes after its binding to ARE. Thus, our results can be supported by this thesis since we have noticed a dose-dependent decrease of enzymes controlled by Nrf2, which may be the result of different levels of ROS. Additionally, many studies [57,58] show that excessive stress can lead to cell death, which can also manifest as inhibition of signaling pathways. The complexity of these processes and their interdependence, in the context of diverse structures of the tested compounds, might suggest the conclusion that increased ROS levels can inhibit the antioxidant activity of Nrf2 through Akt phosphorylation, which stimulates Fox phosphorylation, and consequently leads to the inactivation of Nrf2 and decreased antioxidant enzymes expression [59].

Prolonged Nrf2 activity in cancer cells may promote cancer growth and enhance chemoresistance [60]. Thus, the decreased activation of Nrf2 and an enhanced generation of ROS in cancer cells is highly desirable, and the combination of XN and PEITC seems to act in this way.

Data from in vivo studies suggesting that XN and PEITC synergistically affect animals’ survival, while no effects were observed in the group receiving vehicle and PEITC alone. The observed side effects were a reason to discontinue the experiments according to the ethical guidelines. PEITC is known as a CYP450 isoforms inhibitor, including CYP1A2 [61], and this isoform is also related to XN metabolism [62]. Thus, it is possible that at the tested doses, PEITC may affect XN metabolic pathways and increase the final concentration of XN, which cannot be observed using in vitro model. However, these hypotheses need to be clarified by further studies.

It should be highlighted that, in the presented study, we observed for the first time that XN might interact with the luciferase-luciferin assay. The *Photinus pyralis* luciferase (Fluc) is commonly used for cell-based reporter-gene assays and in vivo xenograft mice models. Fluc catalyzed the oxidation of luciferin to oxyluciferin in the presence of ATP, Mg^2+^, and oxygen, producing light with a peak of 560 nm [63]. The BLI results from in vivo model (both groups receiving XN alone and combination), showed that XN might increase the luminescence signal compared to control. Thus, we performed additional in vitro studies to shed more light on this phenomenon and investigate the type of potential interaction. As we observed, XN added simultaneously with luciferin to PSN-1 did not increase the bioluminescence signal, while cells incubated with XN (at a dose of 5 µM) for 24 h also increased the light intensity (Figure S1).

Several possible mechanisms could explain the interaction of the tested compound with the luciferase-luciferin assay, such as transcriptional activation, posttranscriptional mechanisms, and posttranslational stabilization of reporter enzyme levels [64]. It should be highlighted that the literature data focused mainly on luciferase inhibitors, which may affect firefly luciferase activity by competitive or non-competitive inhibition and non-specific inhibition including denaturation or attenuation of the luminescent signal through photonic processes such as absorbance [65,66,67]. The study performed by Auld and colleagues showed that compounds possessing m-carboxylate group in the structure, such as 3-[5-(2-fluorophenyl)-1,2,4-oxadiazol-3-yl]benzoic acid (PTC124), may inhibit luciferase activity by the interaction of the carboxylate group within the Fluc active site and form a highly potent adenylate adduct [64]. On the other hand, the authors found that PTC124 and its derivatives may also increase cellular Fluc activity levels by posttranslational stabilization [68]. When considering the relatively short luciferase protein half-life (approximately 2–4 h) [69], the compounds which stabilize enzyme and protect against degradation by the protease could overcome the above-mentioned drawback. Furthermore, it was observed that inhibition of luciferase by ATP or luciferin competitive inhibitors might be decreased or diminished by an excess of the substrate [67,68]. Thus, luciferase inhibitors could stabilize the enzyme, while in the presence of luciferin substrate, the inhibitor might compete for the enzyme active site, restore the enzyme activity, and finally increase signal [9]. Our results from in vitro and in vivo models support our hypothesis that XN may affect luciferase-based assay and in consequence increase the bioluminescence signal. However, to explain this phenomenon, further detailed studies must be performed.

## 5. Conclusions

In summary, the present study focused on the combination of phytochemicals, especially the combination of XN and PEITC. We investigated their underlying anti-tumor mechanism in PSN-1 cells for the first time and found that the combination of XN and PEITC induced apoptosis and inhibited the Nrf2, NF-κB and STAT3, and Akt/P70S6K signaling pathways in PSN-1 cancer cells. Furthermore, we also observed that XN and PEITC induced the activation of Nrf2 in non-cancerous MS1 cells, which confirmed their potential protective properties. Undoubtedly, the reason for the different response for studied compounds in cancer and normal cell lines is the level of ROS, whose accumulation can result in cell death. However, due to the complexity and relationship between canonical and non-canonical Nrf2 activation, it is difficult to indicate the exact mechanism by which the combination of XN and PEITC modulates the Nrf2 signaling pathway.

In addition, XN and its combination with PEITC partially suppressed tumor growth in a mice xenograft model. However, further in vivo studies are needed to fully describe XN and PEITC anticancer activity. It should be highlighted that during this study we observed the interaction of XN with the luciferase-luciferin assay for the first time. Our observation opens a new scientific question if this interaction occurs on mRNA level and increases the transcriptional activity or occurs at a protein level via competitive or non-competitive ways that affect enzyme activity.

Taken together, these findings attempt to elucidate the canonical and non-canonical mechanism of modulation of Nrf2 by the combination of XN and PEITC. In fact, the observed biological effects may result from the joint modulation of several key signaling pathways, including the significant inhibition of the NF-κB pathway and the attenuation of JAK/STAT or PI3K pathways by the studied phytochemicals. This may contribute to the future development of XN and PEITC as a new therapeutic agent for cancer treatment.

## Figures and Tables

**Table 1:** The list of the primers used in the real-time PCR.

	Forward Primer	Reverse Primer
Human		
*PBGD*	5′ TCAGATAGCATACAAGAGACC	5′ TGGAATGTTACGAGCAGTG
*TBP*	5′ GGCACCACTCCACTGTATC	5′ GGGATTATATTCGGCGTTTCG
*Nrf2*	5′ ATTGCTACTAATCAGGCTCAG	5′ GTTTGGCTTCTGGACTTGG
*SOD*	5′ CGACAGAAGGAAAGTAATG	5′ TGGATAGAGGATTAAAGTGAGG
*GSTP*	5′ GCAAATACATCTCCCTCATC	5′AGGTTGTAGTCAGCGAAG
*NQO1*	5′ CAATTCAGAGTGGCATTC	5′ GAAGTTTAGGTCAAAGAGG
*Keap1*	5′ ATGGGCGAGAAGTGTGTC	5′ TCTGCTCAGCGAAGTTGG
*p62*	5′ TCTGGGCATTGAAGTTGA	5′ CTCTGTGCTGGAACTCTC
*GSK3β*	5′ ACCCAAATGTAAACTACCAAATG	5′ TCCACGGTCTCCAGTATTAGC
*NF-κB p50*	5′ ATCATCCACCTTCATTCTCAA	5′ AATCCTCCACCACATCTTCC
*NF-κB p65*	5′ CGCCTGTCCTTTCTCATC	5′ ACCTCAATGTCCTCTTTCTG
*COX-2*	5′ CCTGTGCCTGATGATTGC	5′ CAGCCCGTTGGTGAAAGC
Mouse		
*PBGD*	5′ GCCTACCATACTACCTCCT	5′ AAGACAACAGCATCACAAG
*TBP*	5′ TATTGTATCTACCGTGAAT	5′ TAGTCTGGATTGTTCTTC
*Nrf2*	5′ CAGCATAGAGCAGGACAT	5′ TTCGGTATTAAGACACTTAATTC
*SOD*	5′ GGACAAATTACAGGATTA	5′ TTCTTAGAGTGAGGATTA
*NF-κB p50*	5′ CCTCTAGTGAGAAGAACAA	5′ TGACCAACTGAACGATAA
*NF-κB p65*	5′ TACTTGCCAGACACAGATG	5′ GATACTCTTGAAGGTCTCATAGG

**Table 2:** Comparison of the compounds and combinations cytotoxicity in PSN-1 and MS1 cells—IC_50_ values ± SD (µM).

Compound/Mixture	PSN-1		MS1	
	IC_50_	±SD	IC_50_	±SD
I3C	130.0	6.9	>150	
XN	46.0	3.0	49.0	3.0
PEITC	32.0	2.5	35.5	2.4
RVS	112.0	4.2	>150	
XN+I3C	61.0	4.5	67.0	4.5
XN+PEITC	34.5	2.6	38.0	2.2
XN+RVS	59.0	6.0	63.0	4.1
I3C+PEITC	36.0	4.0	41.0	1.5
I3C+RVS	148.0	6.5	>150	
RVS+PEITC	40.0	4.1	41.0	5.0

## Data Availability

Data is contained within the article and Supplementary Materials.

## Supplementary Materials

The following are available online at https://www.mdpi.com/article/10.3390/cells10123556/s1, Figure S1: The effect of xanthohumol on the luminescence signal using in vitro luciferase-luciferin assay; Figure S2. The effect of XN, PEITC, and their combination on the MAPK and PI3K signaling pathways.
