# Supplementary material for: Attenuation of Pancreatic Cancer In Vitro and In Vivo via Modulation of Nrf2 and NF-κB Signaling Pathways by Natural Compounds
Source: Cells. 2021 Dec 16;10(12):3556. doi: 10.3390/cells10123556 (PMC8700195; doi:10.3390/cells10123556)
Supplement: Supplementary file 1 [file cells-10-03556-s001.zip › cells-1434642-supplementary.pdf]

Supplementary Data

Table of contents

Methodology 2

Formulation of Xanthohumol and PEITC for *in vivo* study........ 2

Bioluminescence imaging of PSN-1 cells *in vitro* to investigate the influence of XN on luciferase-luciferin assay. 2

Figure S1. The effect of xanthohumol on the luminescence signal using *in vitro* luciferase-luciferin assay. 3

Results 3

The activation of the MAPK and PI3K signaling pathways 3

Figure S2. The effect of XN, PEITC, and their combination on the MAPK and PI3K signaling pathways. 4

References 4

Methodology

Formulation of Xanthohumol and PEITC for *in vivo* study.

The doses for *in vivo* studies have been selected after careful analysis of available literature data. The dose of Xanthohumol was selected according to Li et al. [1] and Lv et al. [2]. While the dose of phenethyl isothiocyanate was estimated, taking into account data provided by Gao et al. [3] and Ni et al. [4].

Starting from developing a new drug candidate/therapy using the *in vitro* model and moving to a more advanced *in vivo* model, the very significant is the drug formulation for both pharmacodynamic and toxicodynamic evaluations [5]. In general, Xanthohumol, like other phytochemicals, is poorly soluble in water [6], whereas it is well soluble in an organic solvent such as DSMO. However, DMSO should be avoided for *in vivo* studies [7]. Thus, to dissolve tested compounds, we decided to replace DMSO by using ethanol. PEG400 and 0.9% sterile sodium chloride. The solvents used for dissolved tested compounds and the formulation for tested compounds for *in vivo* study were designed according to literature data [8]. The formulation for Xanthohumol, PEITC, and Xanthohumol+PEITC consists of Ethanol 96% : PEG400 : 0.9% Sodium chloride (ratio 1: 2: 3).

Bioluminescence imaging of PSN-1 cells *in vitro* to investigate the influence of XN on luciferase-luciferin assay.

The PSN-1 cells transfected with vector encoding the luciferase gene *luc2* were seeded in Petri dishes (35 mm × 12 mm) at a density of 25 × 10^3 cells/dish. The next day, cells were incubated with XN (at a concentration of 5 μM) or control (vehicle solution). The vehicle solution (Ethanol 96%, PEG400, and 0.9% NaCl) was the same as for *in vivo* studies. The cells were incubated for 24h with the tested compound. The bioluminescence imaging was performed using PhotonImager (BiospaceLab, Nesles-la-Vallée, France) system. Thus, D-luciferin (Goldbio, St Louis, MO, USA) was added at a concentration of 0.15 mg/ml to RPMI-1640 medium, and bioluminescence signal was measured after different time points to monitor signal stabilization. Moreover, we prepared another control to check if there is a direct and immediate interaction between XN and luciferase substrate - luciferin; thus, for two control dishes, we added XN (to have a final concentration of 5 μM) and luciferin at the same time, mixed, and visualized. After imaging, cells were washed twice with PBS (Sigma-Aldrich, St Louis, MO, USA) and lysed using the RIPA buffer (Sigma-Aldrich, St Louis, MO, USA) at 4°C for 30 min. The protein concentration was measured using a BCA assay (ThermoFisher, Waltham, MA, USA) according to the manufacturer's protocol. Bovine albumin (ThermoFisher, Waltham, MA, USA) was used as a standard. Data are presented as a mean value ± SD from two independent experiments and expressed as photon counts per mg of cell protein.

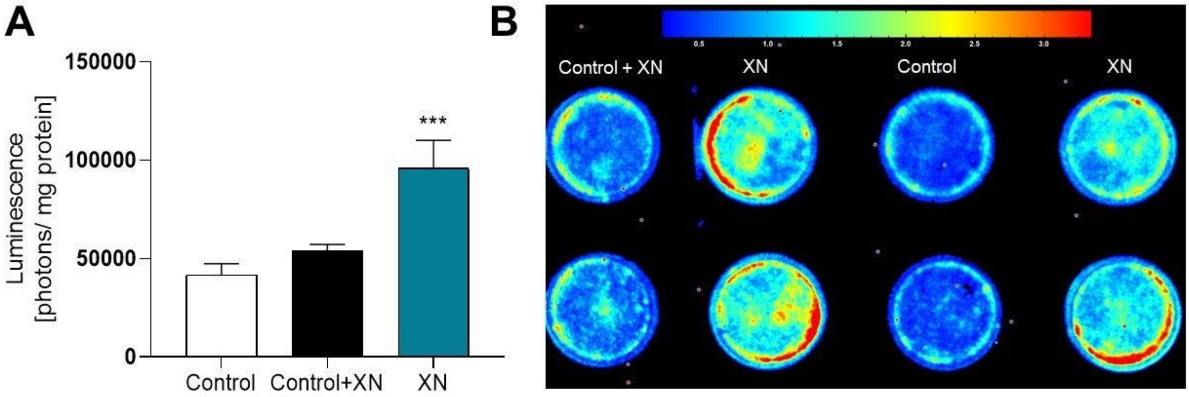

Figure S1. The effect of xanthohumol on the luminescence signal using *in vitro* luciferase-luciferin assay. Panel **A** presents the quantification of luminescence signal in the cells expressed as total photon counts normalized to total cellular protein; panel **B** presents the representative images from the BLI experiment. PSN-1 cells transfected with luciferase vector encoding the luciferase reporter gene *luc2* (*Photinus pyralis*) were seeded at a density of 25×10^3 cells and incubated with 5 µM for 24h. The direct and immediate interaction between the substrate (luciferin), and XN was evaluated by adding XN with luciferin simultaneously for selected dishes (on a graph defined as control+XN). Luminescence signal was measured using PhotonImager. Data are presented as a mean ± SD from two experiments. Statistical analysis was performed using one-way ANOVA, followed by Dunnett's multiple comparisons test. Asterisks indicate the statistical significance, *** $p < 0.0001$.

Results

The activation of the MAPK and PI3K signaling pathways

ERK phosphorylation is the marker of an active state of the MAPK pathway, while phosphorylation of Akt kinase represents PI3K signaling activation. The Muse® MAPK Activation Dual Detection Kit as well as the Muse® PI3K Activation Dual Detection Kit (Merck, Darmstadt, Germany) were used according to the manufacturer's instructions. PMA (0.2 µg/mL) treated cells served as a positive control of the MAPK pathway activation and small molecule compound HS-173 was used as a reference of PI3K signaling attenuation. After 24 h incubation with tested compounds, cells were trypsinized and washed with PBS buffer. After fixation procedure and exposure to permeabilization buffer, cells were incubated for 30 min at room temperature in the dark with a solution of two directly conjugated antibodies against ERK or Akt, targeting phospho-specific and total ERK/Akt-positive cells. Analyses were performed by flow cytometry on Muse® Cell Analyzer, and data were evaluated using Muse® 1.5 Analysis Software.

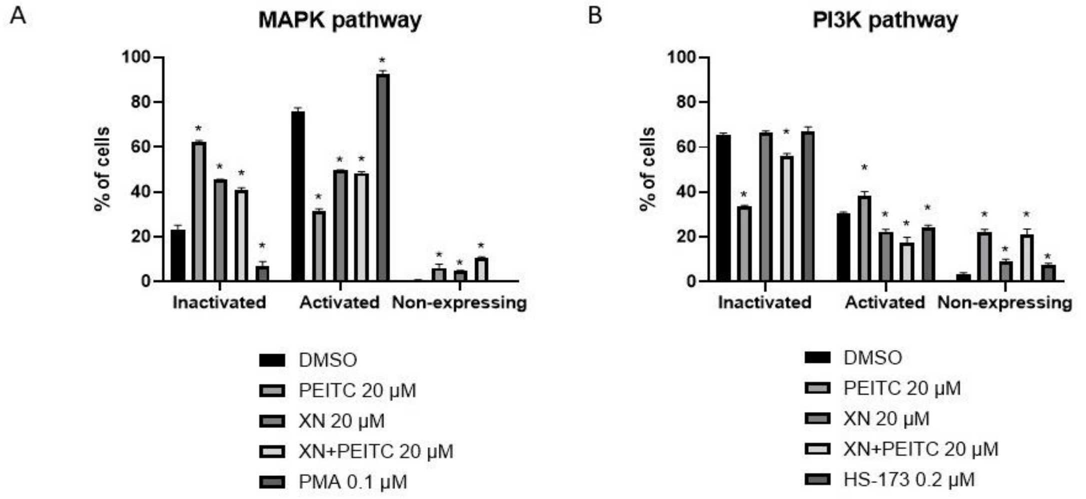

Figure S2. The effect of XN, PEITC, and their combination on the MAPK and PI3K signaling pathways. The effect of XN, PEITC, and their mixture on the activation of MAPK (panel **A**), in terms of ERK phosphorylation as the marker of an active state of MAPK signaling, and PI3K (panel **B**), in terms of Akt kinase phosphorylation as a marker of PI3K signaling in PSN-1 cells. The values are shown as the mean ± SD calculated from three independent experiments (a fold of control). Significance of changes was determined by one-way ANOVA, post-hoc Dunnett's test (* $p < 0.05$).

References

1. Li, F.; Yao, Y.; Huang, H.; Hao, H.; Ying, M. Xanthohumol Attenuates Cisplatin-Induced Nephrotoxicity through Inhibiting NF-KB and Activating Nrf2 Signaling Pathways. Int Immunopharmacol 2018, 61, 277–282, doi:10.1016/j.intimp.2018.05.017.

2. Lv, H.; Liu, Q.; Wen, Z.; Feng, H.; Deng, X.; Ci, X. Xanthohumol Ameliorates Lipopolysaccharide (LPS)-Induced Acute Lung Injury via Induction of AMPK/GSK3β-Nrf2 Signal Axis. Redox Biol 2017, 12, 311–324, doi:10.1016/j.redox.2017.03.001.

3. Gao, N.; Budhraja, A.; Cheng, S.; Liu, E.-H.; Chen, J.; Yang, Z.; Chen, D.; Zhang, Z.; Shi, X. Phenethyl Isothiocyanate Exhibits Antileukemic Activity in Vitro and in Vivo by Inactivation of Akt and Activation of JNK Pathways. Cell Death Dis 2011, 2, e140–e140, doi:10.1038/cddis.2011.22.

4. Ni, W.-Y.; Lu, H.-F.; Hsu, S.-C.; Hsiao, Y.-P.; Liu, K.-C.; Liu, J.-Y.; Ji, B.-C.; Hsueh, S.-C.; Hung, F.-M.; Shang, H.-S.; et al. Phenethyl Isothiocyanate Inhibits in Vivo Growth of Subcutaneous Xenograft Tumors of Human Malignant Melanoma A375.S2 Cells. In Vivo 2014, 28, 891–894.

5. Gad, S.C.; Spainhour, C.B.; Shoemake, C.; Pallman, D.R.S.; Stricker-Krongrad, A.; Downing, P.A.; Seals, R.E.; Eagle, L.A.; Polhamus, K.; Daly, J. Tolerable Levels of Nonclinical Vehicles and Formulations Used in Studies by Multiple Routes in Multiple Species With Notes on Methods to Improve Utility. Int J Toxicol 2016, 35, 95–178, doi:10.1177/1091581815622442.

6. Liu, M.; Hansen, P.E.; Wang, G.; Qiu, L.; Dong, J.; Yin, H.; Qian, Z.; Yang, M.; Miao, J. Pharmacological Profile of Xanthohumol, a Prenylated Flavonoid from Hops (Humulus Lupulus). Molecules 2015, 20, 754–779, doi:10.3390/molecules20010754.

7. Galvao, J.; Davis, B.; Tilley, M.; Normando, E.; Duchen, M.R.; Cordeiro, M.F. Unexpected Low-Dose Toxicity of the Universal Solvent DMSO. FASEB J 2014, 28, 1317–1330, doi:10.1096/fj.13-235440.

8. Saavedra, R.; Rocha, L.B.; Dąbrowski, J.M.; Arnaut, L.G. Modulation of Biodistribution, Pharmacokinetics, and Photosensitivity with the Delivery Vehicle of a Bacteriochlorin Photosensitizer for Photodynamic Therapy. ChemMedChem 2014, 9, 390–398, doi:10.1002/cmdc.201300449.
